# The Influence of BuqiHuoxueTongluo Formula on Histopathology and Pulmonary Function Test in Bleomycin-Induced Idiopathic Pulmonary Fibrosis in Rats

**DOI:** 10.1155/2018/8903021

**Published:** 2018-06-26

**Authors:** Xiaolin Yu, Yanxia Zhang, Xiaohua Yang, Xiaomei Zhang, Xinxiang Wang, Xuemei Liu, Yan Yan

**Affiliations:** ^1^Beijing University of Chinese Medicine, No. 11 on North 3rd Ring Road, Beijing 100029, China; ^2^Department of Respiratory, Dongfang Hospital, Beijing University of Chinese Medicine, No. 6 on 1st District of Fangxingyuan, Beijing 100078, China; ^3^Laboratory Center, Dongfang Hospital, Beijing University of Chinese Medicine, No. 6 on 1st District of Fangxingyuan, Beijing 100078, China

## Abstract

BuqiHuoxueTongluo Formula (BHTF) is an effective herbal prescription based on traditional Chinese medicine for idiopathic pulmonary fibrosis (IPF). The aim of this study was to elucidate the influence of BHTF on induced IPF model through the aspect of histopathology and pulmonary function test. Wistar rats with bleomycin-induced IPF were given BHTF via intragastric gavage. After 14 days and 28 days of treatment, respectively, on these two time points, we first performed pulmonary function test, performed ventilation measure, and traced the Pressure-Volume Loop under anesthesia. Then, rats were sacrificed for hematoxylin-eosin and Masson's trichrome staining, immunohistochemistry staining of TGF-*β*_1_ and *α*-SMA, and observation through transmission electron microscope. BHTF reduced infiltration of inflammation cells, collagen deposition, and fibrosis proliferation in pulmonary mesenchyme, inhibited the expression of TGF-*β*_1_ and *α*-SMA, and avoided the abnormality of ultrastructure and quantities of lamellar bodies. It also ameliorated the parameters of FVC, MVV, PEF, FEF25, and Cdyn, maintained the shape of the Pressure-Volume Loop, and improved hysteresis. BHFT relieved the histopathologic changes, improved ventilation function, compliance, and work of breathing, meliorated the capacity and elasticity of the lungs, and stabilized the alveolar surface tension. Further speaking, it had a potential impact on the secretion of pulmonary surfactant.

## 1. Introduction

Idiopathic pulmonary fibrosis (IPF) is a chronic, diffuse, progressive disease of the pulmonary interstitium with agnogenic etiology [1], and it lacks effective therapy in the clinic now [2]. At present, a glucocorticoid such as prednisone (Pred) and its combination with azathioprine and N-acetylcysteine were widely used to alleviate inflammation, suppress immunoreaction, and relieve symptoms, but it could not reduce the death rate and acute exacerbation [3]. IPF could influence the pulmonary function test of patients, resulting in the decline of Forced Vital Capacity (FVC) [3, 4] and decreased quality of life.

Now, experimental research of IPF commonly induces the model by intratracheal injection of bleomycin (BLM) in rodents [5]. It has been observed in the experimental model induced by BLM that the histopathologic changes of lung tissue were mainly inflammation of alveoli in early stage and then gradual progression of fibrosis deposition [6]. Transforming growth factor *β*_1_ (TGF-*β*_1_) was the most important starting factor of IPF [7]; it could accelerate the progress with which alveolar epithelial cell is transformed into mesenchymal myofibroblast, and then its specific marker *α*-smooth muscle actin (*α*-SMA) was overexpressed [8]. During this course of epithelial-mesenchymal transition (EMT), the function of type II alveolar epithelial cell (AECII) such as secreting pulmonary surfactant (PS) would be influenced because of this transformation [9]. Lamellar body (LB) is an organelle of AECII, which could pack and secrete the pulmonary surfactant [10]; its ultrastructure could reflect the functional status of AECII.

Pulmonary function test could reflect the situation of IPF indirectly. Formerly, research of pulmonary function test with animals mainly concentrated on basic ventilation function and directly measured the compliance of lung tissue [11, 12]. This kind of study had its own limitation, because one side basic ventilation function test showed the characteristic of IPF difficultly, and moreover during a respiratory cycle, the compliance of lung tissue was not invariable [13], and holistic study such as pressure-volume loop of respiratory cycle could reflect the condition of pulmonary function more clearly.

Traditional Chinese medicine (TCM) plays a significant role in the treatment of IPF in China [14]. More and more formulas and herbs were proved to have an effect in preclinical experimental studies of IPF [15]. In the field of TCM, the symptoms of Feibi and Feiwei were described similarly to IPF [16, 17]. The mechanisms included deficiency of Qi, stasis of Xue [18], and obstruction of Luo. With the theory of TCM, this BuqiHuoxueTongluo Formula (BHTF) treats IPF by invigorating Qi, activating Xue, and dredging Luo. We take a further study to reveal the mechanism of BuqiHuoxueTongluo Formula for treating IPF, through the histopathology, immunohistochemistry, and pulmonary function test.

## 2. Materials and Methods

### 2.1. Animals

64 adult male SPF Wistar rats (200 ± 20 g) were provided by Vital River Laboratory Animal Technology Co., Ltd., Beijing, China, and housed in laboratory animal center of Dongfang Hospital, Beijing University of Chinese Medicine. The environmental temperature was controlled at 25 ± 2°C, humidity was maintained at 45%–55%, and circadian rhythm was 12 : 12 h dark/light. All rats were fed with sterilized diet and water. The protocol of experiment was approved by the Ethical Committee for the Experimental Animals at Dongfang Hospital, Beijing University of Chinese Medicine.

### 2.2. Chemicals and Reagents

The compositions and detailed doses of BHTF are shown as follows: Astragali Radix (Huangqi, 30 g), Lonicerae Japonicae Flos (Jinyinhua, 30 g), Angelicae Sinensis Radix (Danggui, 30 g), Glycyrrhizae Radix et Rhizoma (Gancao, 10 g), Dioscoreae Nipponicae Rhizoma (Chuanshanlong, 15 g), Pyrrosiae Folium (Shiwei, 15 g), Fritillariae Thunbergii Bulbus (Zhebeimu, 10 g), Trichosanthis Fructus (Gualou, 15 g), Platycodonis Radix (Jiegeng, 10 g), Aurantii Fructus (Zhiqiao, 10 g), and Rhodiolae Crenulatae Radix et Rhizoma (Hongjingtian, 10 g). The decoction was made by Preparation Center of Dongfang Hospital from crude herbs. After soaking in proper amounts of water for 1 hour, the mixture was boiled for half an hour for extracted solution for the first time. Then, the herbs were boiled for 20 minutes again after soaking for half an hour. The decoction of this was mixed twice together and then further concentrated. The final concentration of decoction was 1 g/ml. The dose of rats was calculated according to the conversion of animal dose to human equivalent doses based on body surface area [19].

Bleomycin was obtained from Nippon Kayaku (Tokyo, Japan). Prednisone was purchased from Lisheng Pharmaceutical Co., Ltd., Tianjin, China. Rabbit antibodies of TGF-*β*_1_ and *α*-SMA were purchased from Sheng-Shi-Zhong-Fang Biotech Co., Ltd., Beijing, China. Pentobarbital sodium and paraformaldehyde were purchased from Hua-Teng-Sheng-Wei Biotech Co., Ltd., Beijing, China.

### 2.3. Experiment Protocol and Model Establishment

After being acclimated for one week, 64 Wistar rats were divided into four groups according to the random number table, 16 cases in each group: control group, BLM group, BLM + Pred group, BLM + BHTF group. Under anesthesia with 40 mg/kg pentobarbital sodium by intraperitoneal injection, 3 mg/kg BLM was injected intratracheally to induce IPF model, and then rats were rotated vertically to distribute BLM more homogeneously in the lung. The next day after establishing the model, rats of BLM + Pred group were given prednisone 3.6 mg/kg/d by intragastric gavage, BLM + BHTF group was treated with a decoction of BuqiHuoxueTongluo Formula 16.2 g/kg/d by intragastric gavage, and control group and BLM group were given the same volume of normal saline per day. On the 14th and 28th day from inducing model, respectively, 8 rats were randomly sacrificed from each group. Before being sacrificed, rats underwent the pulmonary function test under anesthesia.

### 2.4. Lung Histopathology

Lung tissues were fetched on days 14 and 28, respectively, and fixed in 4% paraformaldehyde for 24 h in glass bottles with negative pressure. Then, they were routinely embedded in paraffin and made into 5 *μ*m sliced production, and hematoxylin-eosin and Masson's trichrome staining was performed. Histopathologic score was graded by specialized histopathologists blindly referring to Ashcroft's semiquantitative grading system to divide into 8 grades and described the fibrosis score [20]. The slices stained by Masson's trichrome were picked-up pictures by camera of microscope system and processed by ImagePro plus version 6.0 for Windows. Using the Hue-Saturation-Intensity mode, we selected the area of fibrosis stained by blue and calculated the integral optical density (IOD) for each picture.

### 2.5. Immunohistochemistry

Lung tissues were fixed and embedded routinely, and immunohistochemistry was performed with rabbit polyclonal anti-TGF-*β*_1_ (1 : 100 vol/vol, Abcam) and rabbit monoclonal anti-*α*-SMA (1 : 100 vol/vol, Abcam) as primary antibodies to measure the expression of TGF-*β*1 and *α*-SMA. Tissue sections were deparaffinized and rehydrated successively, and then antigen retrieval was performed by citrate buffer. Slices were incubated with primary antibody (anti-TGF-*β*_1_, anti-*α*-SMA) overnight at 4°C in wet box, rinsed in PBS 3 times, and then incubated with biotinylated goat anti-rabbit IgG and goat anti-mouse IgG secondary antibodies (1 : 100, Abcam). Coloring was performed with the DAB staining method, and then slices were dewaxed and sealed. We observed and photographed the slices using an Olympus microscope. Pictures were analyzed by ImagePro plus version 6.0 for Windows. Using the Hue-Saturation-Intensity mode, we chose positive region stained into brown, and calculated IOD value for each picture.

### 2.6. Pulmonary Function Test

Rats were anesthetized with pentobarbital sodium intraperitoneally (40 mg/kg). After confirming that the rats were in the proper anesthesia state, we opened the skin of the neck by ophthalmic scissors, separated muscles layer by layer, and then exposed the trachea. Subsequently, rats were tracheostomized and the Anires 2005 animal ventilator system (Animal Computer Controlled Pulmonary Function System, Bestlab Co., Ltd., Beijing) was immediately connected. We measured pulmonary function test through two maneuvers: quasistatic pressure ventilation and forced pressure ventilation. Quasistatic pressure ventilation maneuver allowed the measurement of dynamic compliance (Cdyn) and maximal ventilatory volume (MVV). Forced pressure ventilation maneuver measured forced vital capacity (FVC), peak expiratory flow (PEF), and forced expiratory flow at 25% of the FVC exhaled (FEF25). Derived from the data of forced pressure ventilation maneuver, we graphed the pressure-volume loop and calculated the area between the inflation limb and deflation limb, which was reflected the hysteresis. We, respectively, performed these tests on the 14th day and the 28th day.

### 2.7. Transmission Electron Microscope

Lung tissue specimen was fixed by 4% glutaraldehyde, followed by 1% osmic acid postfixation, routinely dehydrated, and embedded in epoxy resin. It was then sliced into ultrathin sections and observed by a transmission electron microscope and images by AMT camera system were saved.

### 2.8. Statistical Analysis

The results were analyzed by SPSS 14.0 software (SPSS Inc., Chicago, USA) and expressed as mean ± standard deviation (SD). Data which were not normally distributed were assessed by Kruskal-Wallis test. Data that complied with normal distribution were assessed by one-way analysis of variance (ANOVA) followed by least significant difference (LSD) test. Value with *P* < 0.05 was considered as a significant difference in statistics.

## 3. Results

### 3.1. BHTF Alleviated the Progression of Fibrosis in BLM-Induced IPF in Rats

**Figure 1:**
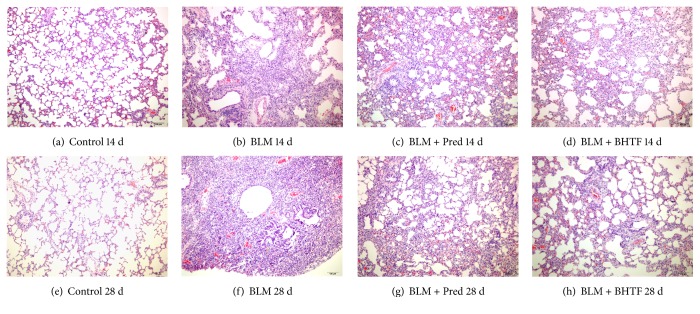
H&E staining of lung tissues (100x). (a) Control group treated by normal saline on day 14. (b) BLM group induced into pulmonary fibrosis and treated by normal saline on day 14. (c) BLM + Pred group treated by prednisone on day 14. (d) BLM + BHTF group treated by prednisone on day 14. (e) Control group treated by normal saline on day 28. (f) BLM group induced into pulmonary fibrosis and treated by normal saline on day 28. (g) BLM + Pred group treated by prednisone on day 28. (h) BLM + BHTF group treated by prednisone on day 28.

H&E staining of lung tissues was performed for observation of inflammation changes [21]. The pictures of H&E stain are shown in Figure 1. Specimen from control group revealed the intactness of bronchi and alveoli structure, no thickness of alveolar septa, and occasional infiltration of inflammation cells in microscope field. No difference existed between day 14 and day 28. Treatment with BLM induced obvious thickness of alveolar septa, destruction of part of bronchi structure, alveolar collapse, proliferation of fibroblast, and infiltration of inflammation cells on day 14. On day 28, the alveolar structure was obliterated by fibrous dyspepsia. Compared with the BLM group, treatment with prednisone and BHTF could reduce the thickness of alveolar septa and infiltration of inflammation cells. Although the progression of pulmonary interstitial changes was inevitable, it was milder in these two groups.

**Figure 2:**
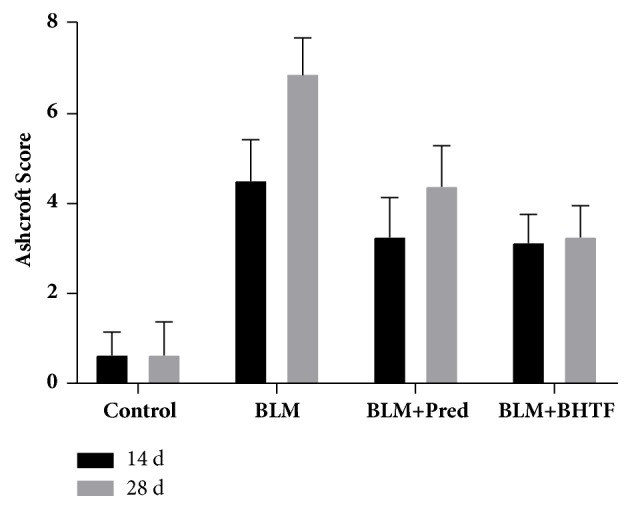
Ashcroft fibrosis score.

Ashcroft fibrosis score is shown in Table 1 and Figure 2. On day 14 and day 28, the fibrosis scores of BLM + Pred group and BLM + BHTF group were lower than BLM group (*P* < 0.05). On day 28, BLM + BHTF group showed lighter grade of fibrosis than BLM + Pred group (*P* < 0.05).

**Figure 3:**
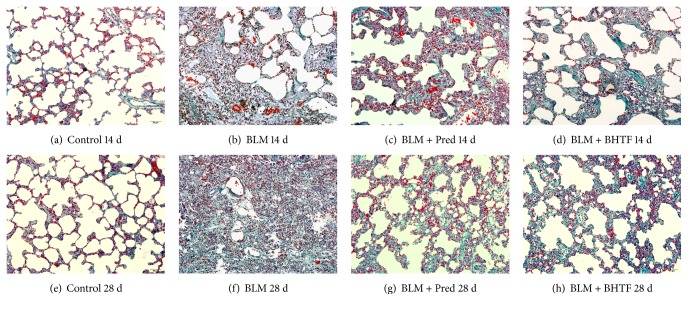
Masson's trichrome staining of lung tissues (200x). (a) Control group treated by normal saline on day 14. (b) BLM group induced into pulmonary fibrosis and treated by normal saline on day 14. (c) BLM + Pred group treated by prednisone on day 14. (d) BLM + BHTF group treated by prednisone on day 14. (e) Control group treated by normal saline on day 28. (f) BLM group induced into pulmonary fibrosis and treated by normal saline on day 28. (g) BLM + Pred group treated by prednisone on day 28. (h) BLM + BHTF group treated by prednisone on day 28.

**Figure 4:**
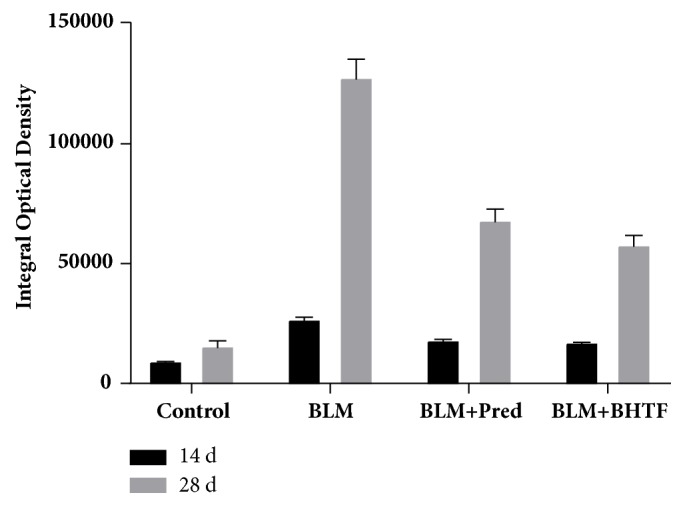
Integral optical density of Masson's trichrome staining.

Lung tissues were stained by Masson's trichrome to evaluate the degree of interstitial fibrosis [21], and the results are illustrated in Figure 3. Control group revealed normal lung tissue structure and slight collagen deposition in the alveolar septa. Treatment with BLM revealed severe collagen deposition, obliteration of interalveolar spaces, and disordered lung structure. Alveolar space on day 28 was almost occluded by fibrosis and normal alveoli were observed scarcely. BLM + Pred group and BLM + BHTF group reduced the destruction to pulmonary interstitium. The IOD of the fibrosis area stained into blue reflected the degree of fibrosis and collagen deposition. As shown in Table 1 and Figure 4, on the two time points, the IOD was significantly lower than BLM group on day 14 and day 28 (*P* < 0.01), and on day 28, BLM + BHTF group showed smaller area stained into blue than BLM + Pred group (*P* < 0.01).

### 3.2. BHTF Reduced the Expression of Indicative Cytokine of BLM-Induced IPF in Rats

**Figure 5:**
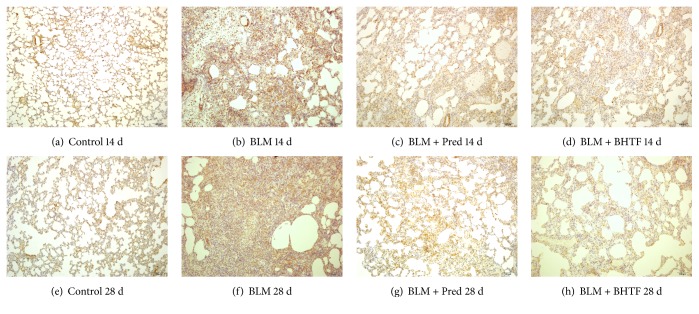
Observation of TGF-*β*_1_ by immunohistochemical staining (100x). (a) Control group treated by normal saline on day 14. (b) BLM group induced into pulmonary fibrosis and treated by normal saline on day 14. (c) BLM + Pred group treated by prednisone on day 14. (d) BLM + BHTF group treated by prednisone on day 14. (e) Control group treated by normal saline on day 28. (f) BLM group induced into pulmonary fibrosis and treated by normal saline on day 28. (g) BLM + Pred group treated by prednisone on day 28. (h) BLM + BHTF group treated by prednisone on day 28.

**Figure 6:**
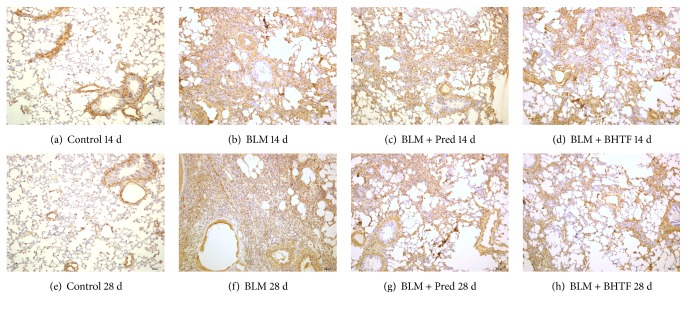
Observation of *α*-SMA by immunohistochemical staining (100x). (a) Control group treated by normal saline on day 14. (b) BLM group induced into pulmonary fibrosis and treated by normal saline on day 14. (c) BLM + Pred group treated by prednisone on day 14. (d) BLM + BHTF group treated by prednisone on day 14. (e) Control group treated by normal saline on day 28. (f) BLM group induced into pulmonary fibrosis and treated by normal saline on day 28. (g) BLM + Pred group treated by prednisone on day 28. (h) BLM + BHTF group treated by prednisone on day 28.

**Figure 7:**
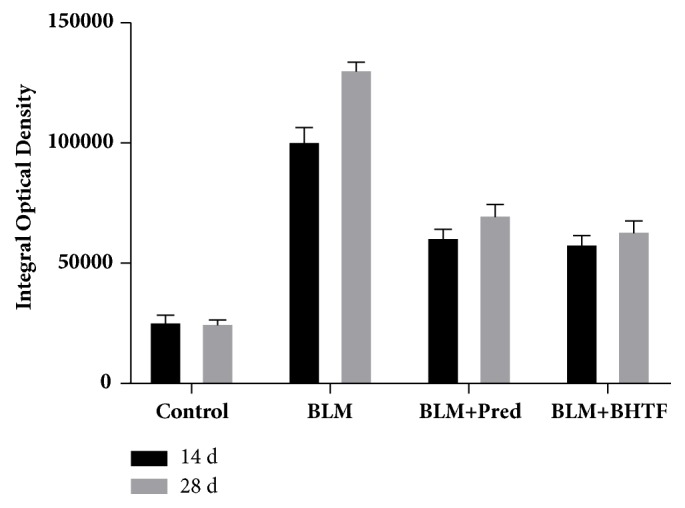
Integral optical density of TGF-*β*_1_.

**Figure 8:**
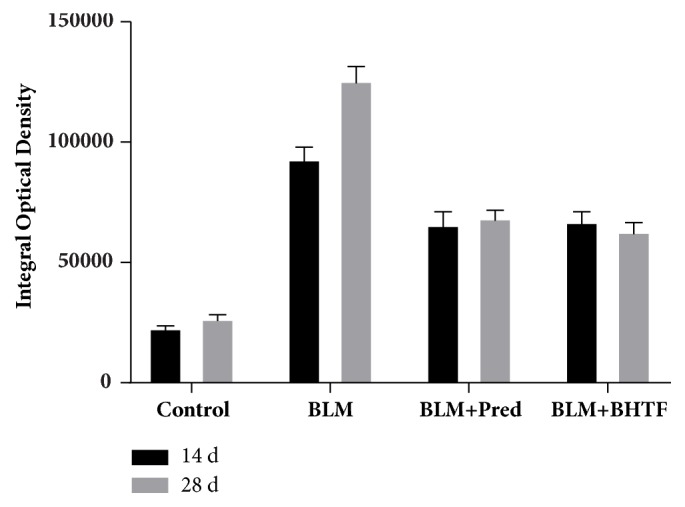
Integral optical density of *α*-SMA.

TGF-*β*_1_ was a generally acknowledged fibrogenic cytokine of IPF [7], and *α*-SMA was an important indicative factor of collagen deposition in pulmonary mesenchyme [8]. The pictures of sections are, respectively, shown in Figures 5 and 6. In normal lung tissue, evidently, immunostaining could be observed in tracheal and vascular wall, and interstitial area was exceedingly weakly positive. Treatment with BLM revealed collagen deposition and fibrosis proliferation. There was a large positive immunolocalization area in pulmonary mesenchyme. The degree of immunostain was evaluated by the IOD of positive area (Table 1, Figures 7 and 8). The IOD value of TGF-*β*_1_ and *α*-SMA was evidently reduced when treated by prednisone and BHTF compared with BLM group whether on day 14 or day 28 (*P* < 0.01). On day 28, the IOD value of BLM + BHTF group was smaller than BLM + Pred group (*P* < 0.05).

### 3.3. BHTF Ameliorated the Ventilation Measure and Dynamic Compliance of BLM-Induced IPF in Rats

**Figure 9:**
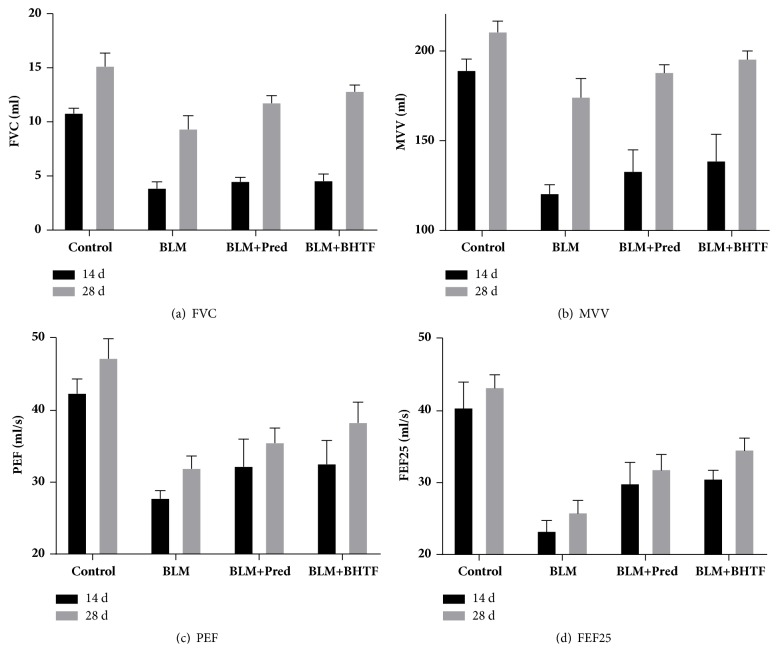
Basic ventilation measure of FVC, MVV, PEF, and FEF25. (a) Pulmonary function test of FVC. (b) Pulmonary function test of MVV. (c) Pulmonary function test of PEF. (d) Pulmonary function test of FEF25.

**Figure 10:**
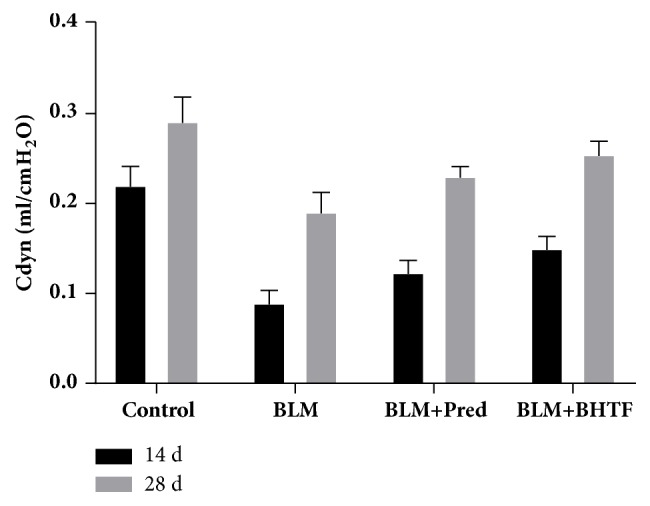
Pulmonary function test of Cdyn.

The basic ventilation measures included FVC, MVV, PEF, and FEF25. As shown in Table 2 and Figures 9(a)–9(d), BLM + Pred group and BLM + BHTF group could ameliorate these ventilation tests compared with the BLM group (*P* < 0.05), and on day 28, treatment by BHTF could ameliorate these ventilation measures comparing with BLM + Pred group. At the two time points of day 14 and day 28 (Table 2, Figure 10), treatment by either prednisone or BHTF could ameliorate Cdyn compared with BLM only (*P* < 0.01), and BLM + BHTF group revealed more remarkable improvement than BLM + Pred group (*P* < 0.05).

### 3.4. BHTF Maintained the Shape of P-V Loop and Ameliorated the Hysteresis of BLM-Induced IPF in Rats

**Figure 11:**
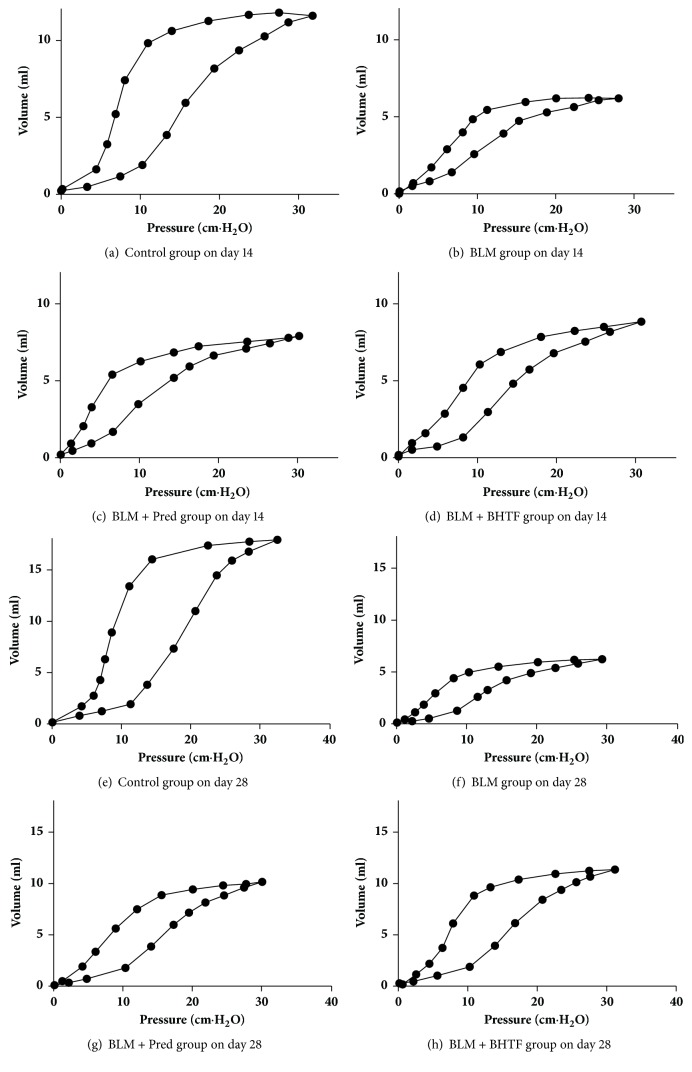
Pressure-volume loops. (a) Pressure-volume loop of control group on day 14. (b) Pressure-volume loop of BLM group on day 14. (c) Pressure-volume loop of BLM + Pred group on day 14. (d) Pressure-volume loop of BLM + BHTF group on day 14. (e) Pressure-volume loop of control group on day 28. (f) Pressure-volume loop of BLM group on day 28. (g) Pressure-volume loop of BLM + Pred group on day 28. (h) Pressure-volume loop of BLM + BHTF group on day 28.

**Figure 12:**
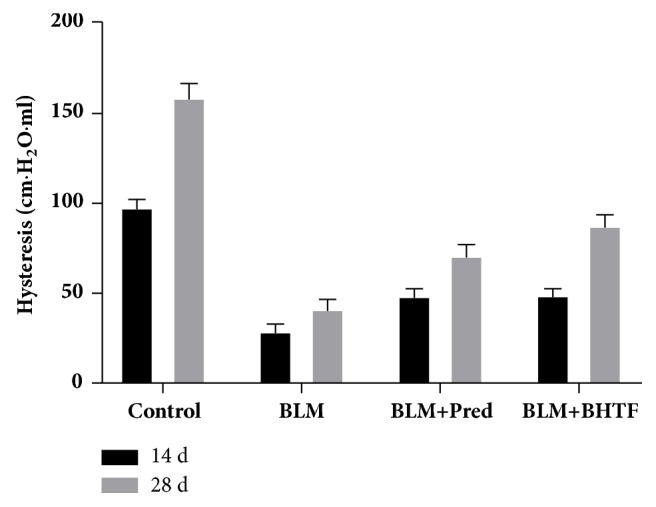
Hysteresis of each group.

The pressure-volume loop reflected the variation of volume along with the lung pressure. On day 14 (Figures 11(a)–11(d)), the lung capacity of BLM group had the narrowest range along with pressure, and the area between inflation and deflation limb was also the smallest in these four groups. BLM + Pred group and BLM + BHTF group had a more preferable lung capacity than BLM group with a wider area between the two limbs. On day 28 (Figures 11(e)–11(h)), the lung capacity and the hysteresis area of these four groups were all increased, but the general shape of each group was still similar to the 14th day's. As shown in Table 2 and Figure 12, hysteresis of each group was enlarged as time goes on, and BLM + BHTF group ameliorated hysteresis clearly compared with BLM + Pred group on day 28 (*P* < 0.01).

### 3.5. BHTF Avoided the Abnormality of Ultrastructure and Quantities of Lamellar Body in BLM-Induced IPF in Rats

**Figure 13:**
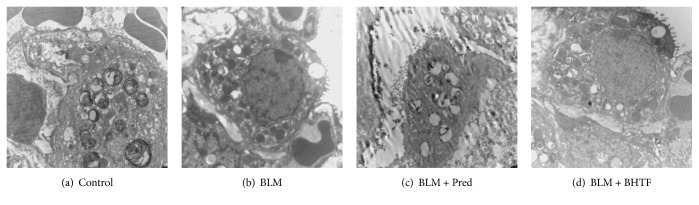
Observation of lamellar body (LB) through TEM (10000x). (a) Control group treated by normal saline. (b) BLM group induced into pulmonary fibrosis and treated by normal saline. (c) BLM + Pred group treated by prednisone. (d) BLM + BHTF group treated by prednisone.

We mainly observed the lamellar body (LB) in AECII by transmission electron microscope on day 28 (Figure 13). LBs in normal lung had numerous quantities, normal structure, and dense arrangement. In BLM group, LBs were swollen and quantities of LBs were decreased. BLM + Pred group and BLM + BHTF group had more LBs than BLM group and the structure was relatively normal.

## 4. Discussion

IPF is a diffuse, fatal disease, and it lacks therapeutic regimens till now [22]. Traditional Chinese medicine has its unique superiority and proper effect in the therapy of IPF [23]. According to the theory of TCM, the early diagnosis of IPF could be considered as Feibi and terminal stage was Feiwei [24], and the pathogenesis is deficiency of Qi, stasis of Xue, and obstruction of Luo [25]. During the clinical practice, the curative effect is obvious by using the therapeutic principle of invigorating Qi, activating Xue, and dredging Luo. Based on these clinical practices, we study the mechanism of BuqiHuoxueTongluo Formula through histopathology and pulmonary function test. All the herbs are measured up to the criteria of the Pharmacopoeia of the People's Republic of China (2015 Edition) and widely used.

Administration of BLM intratracheally is the classical way of model establishment [26]; the histopathologic changes and molecular pathway are similar to human beings [27]. Our histopathologic analysis indicates that, compared with BLM group, the progression of fibrosis in BLM + Pred group and BLM + BHTF group was delayed on day 14 and day 28. BLM + BHTF group revealed lighter degree of fibrosis than BLM + Pred group on day 28. BHTF showed superiority in the long-term therapy of IPF compared with prednisone.

TGF-*β*_1_ is the most important starting factor of IPF [28]; it could promote the process of EMT [29] and facilitate the expression of *α*-SMA [30], which is the specific marker of myofibroblast [31]. On day 14, the expressions of these two factors were both reduced in BLM + Pred group and BLM + BHTF group compared with BLM group, and on day 28, BLM + BHTF group had less expression than BLM + Pred group. This reveals that BHTF could inhibit the release of fibrosis related factors more effectively than prednisone.

For the ventilation measure, FVC reflected the capacity of pulmonary function, and MVV, PEF, and FEF25 comprehensively reflected the flow velocity and pulmonary reserve function. Under the situation of pulmonary fibrosis, BLM group showed significant reduction in pulmonary capacity along with deterioration of airway situation and pulmonary reserve. These revealed the obstruction of airway and abnormality of pulmonary mesenchyme. Combined with the result of Cdyn and histopathologic observation, they revealed that there was irreversible damage of ventilation function and increase of elastic resistance during the experimental pulmonary fibrosis. Treatment with BHTF had an evident mitigation of improving ventilation function and pulmonary compliance; it showed superiority in long-term therapy compared with prednisone.

To reflect the condition of ventilation visually and reveal the mechanical behavior of lung entirely, we need to plot the P-V loop of the whole respiratory cycle, and the slope of this curve is the static compliance of lung tissue [13, 32], which could reflect the elasticity with reduction of airflow resistance compared with dynamic compliance [13]. The P-V loop of control group was nearly “S” shape; the section of both extremities was flatter relatively and middle section was steep. This demonstrated that, in the initial and last stage, the change of lung capacity was small. During the steep section, the capacity changed noteworthily, and the compliance was maximal. The wide range of compliance meant the optimal expansibility of lung tissue. The P-V loop of BLM group was low and flat, the inflection points were not distinct, the steep section was short and slope was small, and the elasticity was limited definitely. Contrasted with BLM group, BLM + Pred group and BLM + BHTF group had a longer and more erect steep section, especially BLM + BHTF group. BHTF could meliorate the capacity and elasticity of lungs more effectively.

During the rhyme of breath, respiratory muscles apply work to overcome frictional resistance and elastic resistance [13], which was called work of breathing (WOB). In P-V loop, WOB could be described as the area between inflation limb and volume axis [33]. In BLM group, based on the ventilation test and compliance measure, the elastic and frictional resistance was significant and it was hard to overcome it for respiratory muscle. Changing unit volume required more pressure, so the P-V loop was low and flat, and the work of breathing was the lowest among these groups. On the contrary, resistance in control group was far lower than BLM group, so WOB was far higher than it. The cause of this was the difference of pulmonary capacity. Supposing the BLM group had the same capacity as control group, its WOB could be significantly higher than others. Treatment with prednisone and BHTF could alleviate the resistance and capacity compared with BLM group and enhanced the WOB.

Comprehensively speaking, during the progression of pulmonary fibrosis, the deterioration of pulmonary function test was along with the changes of histopathologic observation. Destruction and collapse of alveolar structure and thickened alveolar septa resulted in the abnormal resistance and elasticity. So, the P-V loop had varying degrees of changes in each BLM-administered group and decrease of WOB with shallow respiration. Because of the dead space in airway and the lesion of interstitial tissue, the effective ventilation decreased and then influenced the exchange of gas, finally resulting in hypoxia and other complications. And in the long term, BHTF could delay the progression of pulmonary fibrosis and alleviate the pulmonary function.

Meanwhile, we could conclude the surface tension of alveoli through the hysteresis of the P-V loop [34], which is influenced by the pulmonary surfactant [35]. It is a complicated phospholipid and protein complex synthesized and secreted by AECII [10] and packed in LBs [36]. It could maintain the surface tension [37], prevent the hyperinflation and collapse of alveoli, and hold a stable alveoli space. We observed the AECII by TEM and found that the quantity and structure of LBs were changed congruently with the pulmonary function test, which indicates that the IPF could have the mesenchyme changes and meanwhile could influence the surface tension of alveoli. Based on this study, BHTF could alleviate the hysteresis and furthermore perhaps had a potential impact on the secretion of PS in AECII.

## 5. Conclusion

In summary, this study showed that BHTF could reduce the deteriorated histopathologic changes of bleomycin-induced IPF rats, improved ventilation function, compliance, and work of breathing, meliorated the capacity and elasticity of the lungs, and stabilized the alveolar surface tension. Furthermore, based on the P-V loop, we inferred that BHTF had a potential impact on the secretion of PS in AECII and maintained the function of AECII during the course of IPF.

## Figures and Tables

**Table 1:** Histopathologic data of each group on two time points were presented as mean ± SD. Compared with control group ( ^*∗∗*^*P* < 0.01). Compared with BLM group ( ^△^*P* < 0.05). Compared with BLM group ( ^△△^*P* < 0.01). Compared with BLM + Pred group ( ^□^*P* < 0.05). Compared with BLM + Pred group ( ^□□^*P* < 0.01).

Group	Ashcroft Score	IOD of Masson	IOD of TGF-*β*_1_	IOD of *α*-SMA
14 d				
Control	0.62 ± 0.52	8581.13 ± 632.75	24995.82 ± 3563.99	21832.67 ± 1966.80
BLM	4.50 ± 0.93^*∗∗*^	26115.82 ± 1540.87^*∗∗*^	100346.48 ± 6601.19^*∗∗*^	92278.94 ± 6193.47^*∗∗*^
BLM + Pred	3.25 ± 0.89^*∗∗*△^	17258.61 ± 1154.48^*∗∗*△△^	60129.47 ± 4257.64^*∗∗*△△^	64913.49 ± 6535.46^*∗∗*△△^
BLM + BHTF	3.13 ± 0.64^*∗∗*△△^	16475.62 ± 708.46^*∗∗*△△^	57401.83 ± 4382.65^*∗∗*△△^	66209.60 ± 5216.27^*∗∗*△△^
28 d				
Control	0.63 ± 0.74	14762.36 ± 3044.96	24366.61 ± 2182.51	25638.46 ± 2772.93
BLM	6.88 ± 0.83^*∗∗*^	126988.62 ± 8401.09^*∗∗*^	130333.38 ± 4003.75^*∗∗*^	125050.03 ± 7026.05^*∗∗*^
BLM + Pred	4.38 ± 0.92^*∗∗*△△^	67266.04 ± 5606.90^*∗∗*△△^	69582.41 ± 5244.23^*∗∗*△△^	67740.38 ± 4371.74^*∗∗*△△^
BLM + BHTF	3.25 ± 0.71^*∗∗*△△□^	57101.18 ± 4749.87^*∗∗*△△□□^	62756.15 ± 5155.80^*∗∗*△△□□^	62033.93 ± 4921.20^*∗∗*△△□^

**Table 2:** Data of pulmonary function test on two time points were presented as mean ± SD. Compared with the control group ( ^*∗∗*^*P* < 0.01). Compared with BLM group ( ^△^*P* < 0.05). Compared with BLM group ( ^△△^*P* < 0.01). Compared with BLM + Pred group ( ^□^*P* < 0.05). Compared with BLM + Pred group ( ^□□^*P* < 0.01).

Group	FVC (ml)	MVV (ml)	PEF (ml/s)	FEF25 (ml/s)	Cdyn (ml/cmH_2_O)	Hysteresis (cmH_2_O·ml)
14 d						
Control	10.83 ± 0.49	188.91 ± 6.62	42.34 ± 2.04	40.28 ± 3.73	0.22 ± 0.02	96.75 ± 5.55
BLM	3.86 ± 0.62^*∗∗*^	120.39 ± 5.13^*∗∗*^	27.72 ± 1.11^*∗∗*^	23.12 ± 1.62^*∗∗*^	0.09 ± 0.02^*∗∗*^	27.63 ± 5.13^*∗∗*^
BLM + Pred	4.49 ± 0.42^*∗∗*△^	132.72 ± 12.20^*∗∗*△^	32.15 ± 3.86^*∗∗*△△^	29.75 ± 3.08^*∗∗*△△^	0.12 ± 0.01^*∗∗*△△^	47.38 ± 5.15^*∗∗*△△^
BLM + BHTF	4.56 ± 0.65^*∗∗*△^	138.54 ± 15.15^*∗∗*△△^	32.52 ± 3.29^*∗∗*△△^	30.38 ± 1.36^*∗∗*△△^	0.15 ± 0.02^*∗∗*△△^	47.63 ± 4.84^*∗∗*△△^
28 d						
Control	15.18 ± 1.26	210.46 ± 6.21	47.22 ± 2.76	43.14 ± 1.89	0.29 ± 0.03	157.88 ± 8.82
BLM	9.50 ± 1.39^*∗∗*^	174.09 ± 10.53^*∗∗*^	31.88 ± 1.77^*∗∗*^	25.69 ± 1.84^*∗∗*^	0.19 ± 0.02^*∗∗*^	40.25 ± 6.34^*∗∗*^
BLM + Pred	11.78 ± 0.71^*∗∗*△△^	187.73 ± 4.61^*∗∗*△△^	35.46 ± 2.10^*∗∗*△△^	31.72 ± 2.22^*∗∗*△△^	0.23 ± 0.01^*∗∗*△△^	70.00 ± 7.23^*∗∗*△△^
BLM + BHTF	12.62 ± 0.66^*∗∗*△△□^	193.75 ± 5.78^*∗∗*△△□^	38.27 ± 2.88^*∗∗*△△□^	34.43 ± 1.78^*∗∗*△△□□^	0.25 ± 0.02^*∗∗*△△□^	86.63 ± 7.27^*∗∗*△△□□^

## Data Availability

All the data related to this article are described as histopathological pictures and statistical analysis in the manuscript.

## Abbreviations

*α*-SMA: *α*-Smooth muscle actin
AECII: Type II alveolar epithelial cell
ANOVA: One-way analysis of variance
BHTF: BuqiHuoxueTongluo Formula
BLM: Bleomycin
Cdyn: Dynamic compliance
EMT: Epithelial-mesenchymal transition
FEF25: Forced expiratory flow at 25% of the FVC exhaled
FVC: Forced vital capacity
H&E: Hematoxylin-eosin
IOD: Integral optical density
IPF: Idiopathic pulmonary fibrosis
LB: Lamellar body
MVV: Maximal ventilatory volume
PEF: Peak expiratory flow
Pred: Prednisone
PS: Pulmonary surfactant
P-V loop: Pressure-volume loop
SD: Standard deviation
TCM: Traditional Chinese medicine
TEM: Transmission electron microscope
TGF-*β*_1_: Transforming growth factor *β*_1_
WOB: Work of breathing.

## Supplementary Materials

Supplementary Materials Ashcroft's semiquantitative grading system.

## References

[1] Malaviya R., Laskin J. D., Laskin D. L. (2017). Anti-TNF*α* therapy in inflammatory lung diseases. *Pharmacology & Therapeutics*.

[2] Li F.-Z., Cai P.-C., Song L.-J. (2015). Crosstalk between calpain activation and TGF-*β*1 augments collagen-I synthesis in pulmonary fibrosis. *Biochimica et Biophysica Acta (BBA) - Molecular Basis of Disease*.

[3] Raghu G., Rochwerg B., Zhang Y., etal. (2015). An official ATS/ERS/JRS/ALAT clinical practice guideline: treatment of idiopathic pulmonary fibrosis. An update of the 2011 clinical practice guideline. *American Journal of Respiratory And Critical Care Medicine*.

[4] Canestaro W. J., Forrester S. H., Raghu G., Ho L., Devine B. E. (2016). Drug treatment of idiopathic pulmonary fibrosis systematic review and network meta-analysis. *CHEST*.

[5] Hoymann G H., Schaudien D., Hansen T. (2016). B62 the biology of scarring. where are we now: functional, histological and biochemical endpoints for assessing antifibrotic efficacy in a rat model of pulmonary fibrosis. *American Thoracic Society*.

[6] O’Reilly P. J., Ding Q., Akthar S. (2017). Angiotensin-converting enzyme defines matrikine-regulated inflammation and fibrosis. *JCI Insight*.

[7] Mora A. L., Rojas M., Pardo A., Selman M. (2017). Emerging therapies for idiopathic pulmonary fibrosis, a progressive age-related disease. *Nature Reviews Drug Discovery*.

[8] Kim K. K., Kugler M. C., Wolters P. J. (2006). Alveolar epithelial cell mesenchymal transition develops *in vivo* during pulmonary fibrosis and is regulated by the extracellular matrix. *Proceedings of the National Acadamy of Sciences of the United States of America*.

[9] Cherubini E., Mariotta S., Scozzi D. (2017). BDNF/TrkB axis activation promotes epithelial-mesenchymal transition in idiopathic pulmonary fibrosis. *Journal of Translational Medicine*.

[10] De Aguiar Vallim T. Q., Lee E., Merriott D. J. (2017). ABCG1 regulates pulmonary surfactant metabolism in mice and men. *Journal of Lipid Research*.

[11] Guan R., Zhao X., Wang X. (2016). Emodin alleviates bleomycin-induced pulmonary fibrosis in rats. *Toxicology Letters*.

[12] Wuyts W. A., Willems S., Vos R. (2010). Azithromycin reduces pulmonary fibrosis in a bleomycin mouse model. *Experimental Lung Research*.

[13] Zhu L., Cai Y. Y., Niu S. F. (2014). *Clinical Pulmonary Function*.

[14] Zhang Y., Mao X., Su J. (2017). A network pharmacology-based strategy deciphers the underlying molecular mechanisms of Qixuehe Capsule in the treatment of menstrual disorders. *Chinese Medicine*.

[15] Yang J., Cui Y., Kolb M. (2009). How useful is traditional herbal medicine for pulmonary fibrosis?. *Respirology*.

[16] Chen F., Wang P.-L., Fan X.-S., Yu J.-H., Zhu Y., Zhu Z.-H. (2016). Effect of Renshen Pingfei Decoction, a traditional Chinese prescription, on IPF induced by Bleomycin in rats and regulation of TGF-*β*1/Smad3. *Journal of Ethnopharmacology*.

[17] Li M., Li Y., Li J. (2017). long-term effects of TCM Yangqing Kangxian formula on bleomycin-induced pulmonary fibrosis in rats via regulating nuclear factor-*κ*B Signaling. *Evidence-Based Complementary and Alternative Medicine*.

[18] Huang Y. J. (2017). *Data Mining of Traditional Chinese Medicine in Treatment Treating Pulmonary Fibrosis and related intervening mechanisms study of Yangfei Huoxue Prescription*.

[19] Food and Drug Administration (2005). Guidance for industry: estimating the maximum safe starting dose in initial clinical trials for therapeutics in adult healthy volunteers. *Center for Drug Evaluation and Research (CDER)*.

[20] Ashcroft T., Simpson J. M., Timbrell V. (1988). Simple method of estimating severity of pulmonary fibrosis on a numerical scale. *Journal of Clinical Pathology*.

[21] Liu Y., Lu F., Kang L., Wang Z., Wang Y. (2017). Pirfenidone attenuates bleomycin-induced pulmonary fibrosis in mice by regulating Nrf2/Bach1 equilibrium. *BMC Pulmonary Medicine*.

[22] King T. E., Pardo A., Selman M. (2011). Idiopathic pulmonary fibrosis. *The Lancet*.

[23] Huang H., Peng X., Zhong C. (2013). Idiopathic pulmonary fibrosis: The current status of its epidemiology, diagnosis, and treatment in China. *Intractable and Rare Diseases Research*.

[24] Huang Y. J., Gong J. N. (2016). Analysis on pulmonary fibrosis of name, pathogenesis, sydromes and treatment. *Journal of Liaoning University of Traditional Chinese Medicine*.

[25] Liu L. J., Sun F. F. (2017). Yiqi Huoxue Tongluo method in the treatment of idiopathic pulmonary fibrosis. *Shanxi University of Traditional Chinese Medicine*.

[26] Chaudhary N. I., Schnapp A., Park J. E. (2006). Pharmacologic differentiation of inflammation and fibrosis in the rat bleomycin model. *American Journal of Respiratory and Critical Care Medicine*.

[27] Moore B. B., Hogaboam C. M. (2008). Murine models of pulmonary fibrosis. *American Journal of Physiology-Lung Cellular and Molecular Physiology*.

[28] Yamazaki R., Kasuya Y., Fujita T. (2017). Antifibrotic effects of cyclosporine A on TGF-*β*1–treated lung fibroblasts and lungs from bleomycin-treated mice: role of hypoxia-inducible factor-1*α*. *The FASEB Journal*.

[29] Kalluri R., Weinberg R. A. (2009). The basics of epithelial-mesenchymal transition. *The Journal of Clinical Investigation*.

[30] Lamouille S., Xu J., Derynck R. (2014). Molecular mechanisms of epithelial-mesenchymal transition. *Nature Reviews Molecular Cell Biology*.

[31] Willis B. C., Borok Z. (2007). TGF-*β*-induced EMT: mechanisms and implications for fibrotic lung disease. *American Journal of Physiology-Lung Cellular and Molecular Physiology*.

[32] Salazar E., Knowles J H. (1964). An analysis of pressure-volume characteristics of the lungs. *Journal of applied physiology*.

[33] Phillips J. E., Peng R., Burns L. (2012). Bleomycin induced lung fibrosis increases work of breathing in the mouse. *Pulmonary Pharmacology and Therapeutics*.

[34] Harris R. S. (2005). Pressure-volume curves of the respiratory system. *Respiratory Care*.

[35] Lopez-Rodriguez E., Laukamp C., Hidalgo A., etal. (2016). Using pulmonary surfactant as pirfenidone vehicle to target lung epithelium in bleomycin-induced lung fibrosis. *Lung Fibrosis: New Directions to Inform The Future. American Thoracic Society*.

[36] Kononenko V., Erman A., Petan T. (2017). Harmful at non-cytotoxic concentrations: SiO2-SPIONs affect surfactant metabolism and lamellar body biogenesis in A549 human alveolar epithelial cells. *Nanotoxicology*.

[37] Echaide M., Autilio C., Arroyo R., Perez-Gil J. (2017). Restoring pulmonary surfactant membranes and films at the respiratory surface. *Biochimica et Biophysica Acta (BBA) - Biomembranes*.

