# Supplementary material for: The Influence of BuqiHuoxueTongluo Formula on Histopathology and Pulmonary Function Test in Bleomycin-Induced Idiopathic Pulmonary Fibrosis in Rats
Source: Evid Based Complement Alternat Med. 2018 Jun 26;2018:8903021. doi: 10.1155/2018/8903021 (PMC6038586; doi:10.1155/2018/8903021)
Supplement: Supplementary Materials — Ashcroft's semiquantitative grading system. [file 8903021.f1.pdf]

Ashcroft's semi-quantitative grading system

Grade 0, normal lung;

Grade 1, minimal fibrous thickening of alveolar or bronchiolar walls;

Grade 2-3, moderate thickening of walls without obvious damage to lung architecture;

Grade 4-5, increased fibrosis with definite damage to lung structure and formation of fibrous bands or small fibrous masses;

Grade 6-7, severe distortion of structure and large fibrous areas; "honeycomb lung" was placed in this category;

Grade 8, total fibrous obliteration of the field.

Ashcroft T, Simpson J M, Timbrell V. Simple method of estimating severity of pulmonary fibrosis on a numerical scale[J]. Journal of clinical pathology, 1988, 41(4): 467-470.
